# Effect of l-arginine compared to placebo on sexual function in women with major depressive disorder: a randomized controlled trial

**DOI:** 10.1186/s12888-024-05781-5

**Published:** 2024-05-14

**Authors:** Pouria Torkaman, Azadeh Mazaheri Meybodi, Ali Kheradmand, Shiva Eiliaei, Maria Tavakoli Ardakani

**Affiliations:** 1 Department of Clinical Pharmacy, School of Pharmacy, Shahid Beheshti University of Medical Sciences, Tehran, Iran; 2 Department of Psychiatry, Taleghani Hospital Research Development Committee, School of Medicine, Shahid Beheshti University of Medical Sciences, Tehran, Iran

**Keywords:** Arginine, L-arginine, Sexual dysfunction, Major depressive disorder, Selective serotonin reuptake inhibitors, SSRIs

## Abstract

**Background:**

While some evidence suggests that l-arginine may improve sexual function and alleviate depression, it has not been investigated in women with depression to assess both its effects on the depression and sexual function concurrently.

**Methods:**

Patients who had received a diagnosis of major depressive disorder, as determined by predetermined inclusion and exclusion criteria, were enrolled in this triple-blind clinical trial. Patients were divided into two groups: group A, received L-arginine 1 gram twice daily, and group B, received a placebo for four weeks. They were evaluated at baseline, after four and eight weeks with the Hamilton Depression Rating Scale (HDRS), and Rosen’s questionnaire or Female Sexual Function Index (FSFI).

**Results:**

A decrease in the severity of depression was observed in all patients, which was determined due to Hamilton’s questionnaire (P-value < 0.001). During the time in group A, FSFI increased. Based on the FSFI questionnaire, they had improvement in some domains, including the lubrication index and orgasm index, which significantly changed in the eighth week compared to the baseline (P-value < 0.05). However, these two indicators did not change statistically significantly compared to the placebo group.

**Conclusion:**

L-arginine supplementation can improve sexual function, particularly lubrication and orgasm, and mood in women with depression, with minimal side effects observed. Additional research is necessary to validate these results by examining the effects of higher dosages, extended durations, and larger populations of depressed patients.

**Trial registration:**

Iranian Registry of Clinical Trial: IRCT20100127003210N26.

## Background

Research showed that sexual function is one of the key factors affecting quality of life. This is further supported by studies where nearly half of participants explicitly mentioned its importance to their overall well-being. This relationship is bidirectional; the people with better physical health are more satisfied with their sexual function [1]. Based on the definition of World Health Organization, sexual health can be expressed as follows: “…a state of physical, emotional, mental and social well-being about sexuality; it is not merely the absence of disease, dysfunction or infirmity” [2]. An imperative aspect to bear in mind regarding sexual function is that improper sexual intercourse among partners has the potential to undermine various facets of their relationship [3]. Various methods are used to improve sexual function, and many studies were conducted and are being conducted in this field. The number and types of these studies indicate the importance of this issue among different societies. Studies on the nutrition [4], exercise [5], complementary medicine [6], and even stimulants [7] are among these studies. Contrary to old beliefs, sexual disorders are not exclusive to men, and the prevalence of these disorders in the women is high. This prevalence differs in multiple societies, but this difference does not mean these disorders are minor [8, 9]. The estimated prevalence of sexual problems in women between 2000 and 2019 by data from 21 eligible studies of sexual disorders was 50.75% based on the systematic review and meta-analysis. Furthermore, the prevalence of pain and disorders in arousal, sexual desire, lubrication, orgasm, and sexual satisfaction were calculated as 39.08%, 48.21%, 50.70%, 37.60%, 40.16%, and 35.02%, respectively [10]. One of the influential factors in sexual function, and the occurrence of sexual disorders is the presence of underlying diseases, such as psychiatric disease, especially major depressive disorder [11]. The prevalence of sexual disorders in the patients with major depressive disorder has been reported differently. In some studies, this disorder was reported in nearly half of patients with major depressive disorder [12, 13]. A meta-analysis in 2022 showed that sexual dysfunction is prevalent in females with depressive disorders who are not in the pharmacological treatment. Women with major depressive disorder showed high rates of sexual impairment, including 47.22% for arousal, 65.30% for desire, 36.98% for lubrication, 34.17% for orgasm, and 33.91% for sexual satisfaction [14]. There is still a need for safe, affordable, and easily available remedies, even in cases when women’s sexual problems can be treated with certain pharmaceutical approaches [15, 16]. However, certain antidepressants can cause sexual dysfunction, making it more challenging to address sexual issues in patients also dealing with major depressive disorder [17]. The development of sexual dysfunction following selective serotonin reuptake inhibitor medications was confirmed in women before [18, 19]. This disorder can be seen in different fields, including sexual desire, difficulty in arousal, and anorgasmia [20, 21]. For this reason, one of the most critical challenges to treat depressed patients with selective serotonin reuptake inhibitors is the sexual disorders induced by these medications, and there are few options for managing this complication [22]. It should be mentioned that this problem is one of the primary causes of medication non-adherence in patients with depression [23]. According to some research, L-arginine may improve sexual function and have a positive impact on the cellular and molecular processes underlying Major Depressive Disorder [24–26]. Based on some animal studies, using L-arginine has reduced depression and related biological signals [27, 28]. Nitric oxide synthase is involved in the production of nitric oxide. Nitric oxide is one of the metabolites of L-arginine, which has many activities in the body, including its role in sexual functions [29]. In the women with sexual dysfunction, a much lower level of nitric oxide synthase was reported in the epithelium of the female genital tract compared to women without sexual dysfunction, which means decreased nitric oxide in those patients [30]. .

Previous studies on the effectiveness of L-arginine in treating sexual disorders in men were conducted, and promising results reported [31–33].

The safety of long-term use of L-arginine, was confirmed in some studies and no specific side effects were observed in the consumption of L-arginine up to doses of 30 g per day for 90 days [34]. The main goal of the current study was the evaluation of the effect of L-arginine on women suffering from depression in terms of its impact on mood and sexual function. Furthermore, its safety in these patients were evaluated.

## Materials and methods

**Fig. 1:**
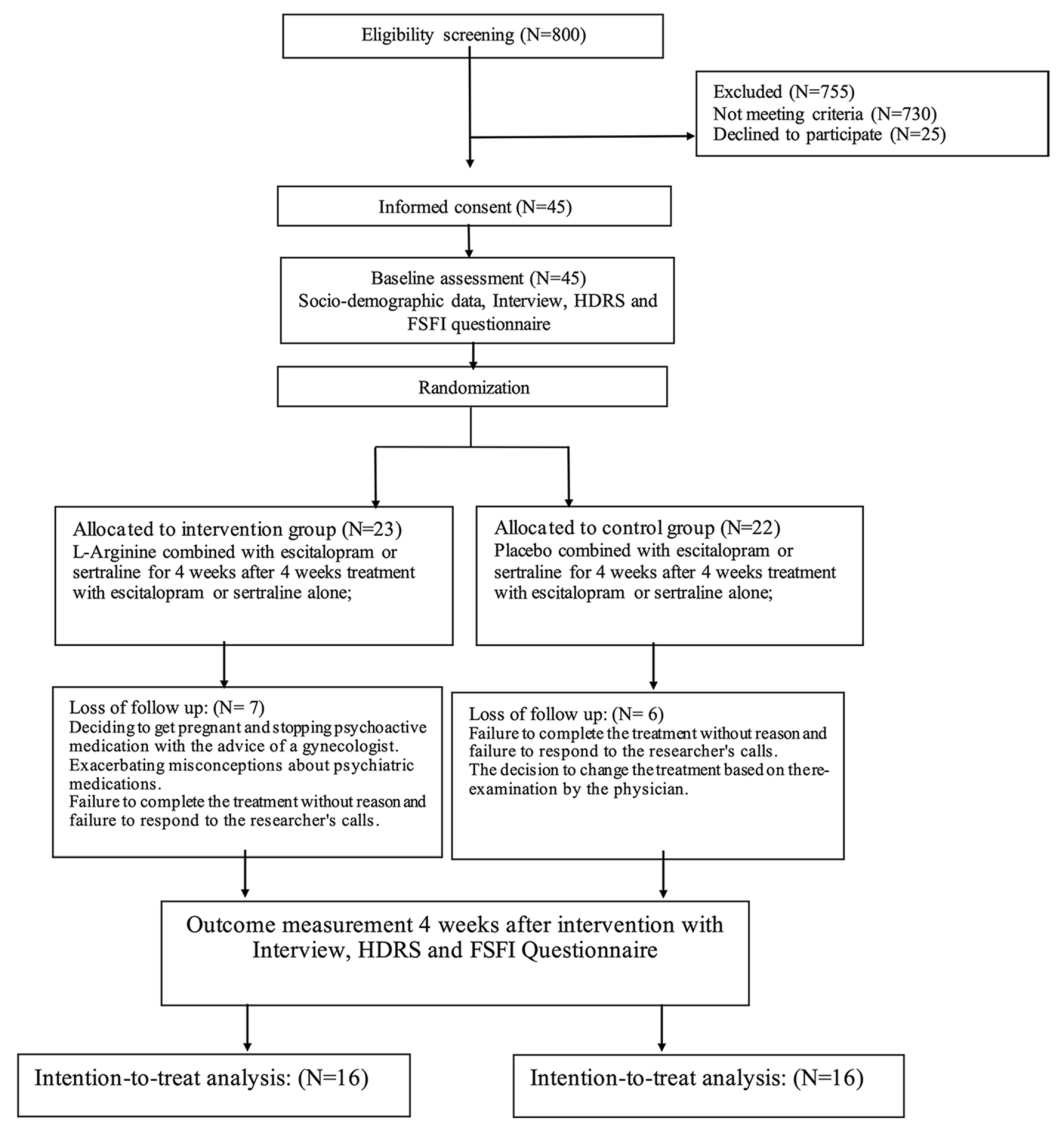
Flowchart of the recruitment of volunteers and allocation to each group of intervention

This is a triple-blind randomized clinical trial study to evaluate the effectiveness of adding L-arginine to the treatment regimen of female patients with major depressive disorder on sexual function. The study’s patients were randomized by blocking www.sealedenvelope.comwebsite. Regarding the sample size, according to Mojdeh and Mohammadi’s study of Iranian women’s sexual function [35], the standard deviation value measured for FSFI was considered equal to 5.1. 32 patients were included in the study for all of the two groups, based on the assumption that the ideal difference between the two groups in this variable is equal to 5, alpha is equal to 0.05, and power is equal to 80%. The number of patients for each group was determined to be 16. The placebo and L-arginine groups had 16 people (See Fig. 1). To evaluate the severity of depression of patients included in the study, Hamilton’s depression questionnaire [36] was used, and Rosen’s questionnaire or FSFI was used to assess their sexual function [37].

The time and place of this study were from August 2022 to August 2023 in the psychiatric departments of Ayatollah Taleghani Hospital, in Tehran, Iran. The inclusion criteria were: (1) Female patients in the age group of 18–60 years old; (2) the patients with major depressive disorder based on DSM-5 diagnostic criteria, (3) No history of taking anti-depressant medication in the last month before entering the study; (4) Absence of anatomical disorder in the genital system; (5) Ability to take oral medications; (6) Lack of active ulcers in the gastrointestinal tract; (7) Having partner during the study period.

The exclusion criteria were as follows: (1) History of diabetes mellitus; (2) Impaired blood pressure based on AHA 2022 clinical guidelines that ˃ 140/90 or taking nitrate-based drugs; (3) Taking potassium-sparing medication; (4) Taking medicines that affect the patient’s sexual function in any way or substances or alcohol that affects sexual function; (5) Suffering from other neurological and mental disorders in addition to suffering from major depressive disorder; (6) Patients who have recently had a heart attack; (7) Unstable marital relationship in such a way that sexual relations between couples are damaged.

L-Arginine were used as a tablet 1 g twice daily (Made by Jalinous Pharmaceutical Company in Iran). Hamilton’s questionnaire was used to evaluate patients’ depression. This questionnaire has 21 items, the first 17 of which are used to assess the severity of depression. In this questionnaire, the scores of patients are interpreted as follows based on the following ranges: Between 0 and 7: absence of depression; 8 to 13: mild depression; 14 to 18: moderate depression; 19 to 22: severe depression; 23 and above: very severe depression; Rosen’s Questionnaire, or FSFI, was used to evaluate women’s sexual function. This questionnaire has 19 items and evaluates six domain areas: desire, psychological stimulation, moisture, orgasm, satisfaction, and pain. The minimum score in this questionnaire is 2, and the maximum score is 30. The higher score, is associated with better sexual function.

Patients with major depressive disorder were first prescribed one of the two medications, sertraline or escitalopram, at a starting dosage of 10 mg or 50 mg, respectively. Then, based on the patient’s response and tolerance, the desired dose of each of these two medications was increased to the maximum approved dose which means: Sertraline: 200 mg/day and Escitalopram: 20 mg/day.

After four weeks, the patients were divided into two placebo or L-arginine groups along with the standard antidepressant treatment, which included one of two medications, including escitalopram or sertraline. The duration of L-arginine use was four weeks. Patients were followed up through phone calls, pill counting, and examination in the clinic. This follow-up included assessing medication use, side effects and drug interactions, completing Hamilton’s depression questionnaire, and Rosen’s questionnaire or FSFI and patients’ histories. The mentioned questionnaires were completed three times, which are as follows: at the beginning of the diagnosis of major depressive disorder and before starting the treatment with SSRIs, in the fourth week after the patient entered the study, and before adding L-arginine or placebo; and in the eighth week that means after four weeks of taking L-arginine or placebo in addition to the standard antidepressant treatment; Besides, considering one of the secondary goals of this study was to evaluate the side effects of L- arginine consumption, by asking of the patients during the entire treatment period, by phone or during clinic visits.

## Statistical analysis

The normality of quantitative data was measured using Shapiro-Wilk test. Quantitative data are described using mean and standard deviation, and qualitative data are represented as frequency and percentage. The parametric t-test or the nonparametric Mann-Whitney U test was employed to examine the differences in the means of the quantitative variables between the two groups. The Levene’s test assessed the validity of the normal distribution assumption on the equality of variances in quantitative data. The chi-square test or Fisher’s exact test was used to check the difference in the distribution of grouped variables. Data were analyzed according to the initial group allocation (intention to treat). To study the changes in HDRS index and indices related to FSFI after the intervention compared to before (comparison of the period before and after), a parametric test (paired t-test), and nonparametric test (Wilcoxon test) were used according to the normality of the variables. Regarding the non-acceptance of the assumptions of repeated measures analysis according to the available data, the regression test of linear generalized estimating equations (GEE) was used to investigate the trend of repeated quantitative variables over time. The correlation structure approach in the GEE linear model has been exchangeable. Finally, the univariate and multiple GEE model with the exchangeable correlation structure approach was used to study the effect of the intervention in the presence of confounding variables. All analyses were performed at a significance level of less than 0.05 using STATA software version 14.

## Results

Thirty-two people were included, 16 in the placebo group and 16 in the L-arginine group (Fig. 1).

Demographic characteristics and clinical assessment of the participants are shown in (Table 1). The condition of the patients in the two areas of depression intensity and sexual function at the beginning of study is determined (Table 2).

**Table 1:** Demographic and clinical assessment of participants between interventional groups

Variables	Placebo (*n* = 16)	L-arginine (*n*= 16)	Total (*n*= 32)	P_value
**Demographic characteristics**				
**Age (years) (Mean ± SD)**	40.5 ± 12.2	40.1 ± 1.5	40.3 ± 9.5	0.913
**Place of living**				
Capital N(%)	14 (87.50)	9 (56.25)	23 (71.88)	0.113
Other N(%)	2 (12.50)	7 (43.75)	9 (28.13)	
**Level of education**				
Illiterate N(%)	1 (6.25)	1 (6.25)	2 (6.25)	0.455
Primary school N(%)	3 (18.75)	4 (25.00)	7 (21.88)	
High school degree N(%)	2 (12.50)	6 (37.50)	8 (25.00)	
Diploma N(%)	2 (12.50)	1 (6.25)	3 (9.38)	
University degree N(%)	8 (50.00)	4 (25.00)	12 (37.50)	
**Job status**				
Employed N(%)	7 (43.75)	5 (31.25)	12 (37.50)	0.465
Unemployed N(%)	9 (56.25)	11 (68.75)	20 (62.50)	
**Anthropometric assessment**				
Weight (kg) (Mean ± SD)	66.1 ± 10.2	74.4 ± 11.7	70.3 ± 11.6	0.042^*^
Height (cm) (Mean ± SD)	162.1 ± 7.4	161.5 ± 5.1	161.8 ± 6.3	0.764
Body Mass Index (BMI, kg/cm^2^) (Mean ± SD)	25.2 ± 4.3	28.5 ± 4.6	26.9 ± 4.7	0.046^*^
**Medical history**				
Without comorbidity N (%)	14 (87.50)	12 (75.00)	6(18.76)	
With comorbidity N (%)	2 (12.5)	4 (25)	26.93 ± 4.73	0.862
**Hamilton Depression Rating Scale (HDRS)**				
Baseline HDRS (Mean ± SD)	15.06 ± 7.05	18.06 ± 8.03	16.56 ± 7.59	0.57
**Female Sexual Function Index (FSFI)**				
Baseline FSFI (Mean ± SD)	22.71 ± 8.98	17.78 ± 10.96	20.24 ± 10.17	0.18
**FSFI items**				
Baseline Desire (Mean ± SD)	3.67 ± 2.58	3.00 ± 1.33	3.33 ± 2.04	0.581
Baseline Arousal (Mean ± SD)	3.15 ± 1.87	2.63 ± 2.10	2.89 ± 1.97	0.494
Baseline Lubrication (Mean ± SD)	3.90 ± 1.76	2.92 ± 2.23	3.41 ± 2.04	0.183
Baseline Orgasm (Mean ± SD)	3.90 ± 1.91	2.63 ± 2.14	3.26 ± 2.10	0.09
Baseline Satisfaction (Mean ± SD)	3.81 ± 1.88	3.35 ± 2.04	3.58 ± 1.94	0.51
Baseline Pain (Mean ± SD)	4.27 ± 1.97	3.23 ± 2.39	3.75 ± 2.22	0.293

Statistically significant, *p*_value < 0.05

**Table 2:** Assessment of depression using Hamilton Scale (HDRS) between two intervention groups

Variables	Placebo	L-arginine	Total	P_value
	(*n* = 16)	(*n*= 16)	(*n*= 32)	
**Hamilton Depression Rating Scale (HDRS)**				
Baseline HDRS N (%)				
Normal	0 (0.00)	0 (0.00)	0 (0.00)	
Mild	9 (56.25)	6 (37.50)	15 (46.88)	
Moderate	3 (18.75)	4 (25.00)	7 (21.88)	0.811
Severe	1 (6.25)	1 (6.25)	2 (6.25)	
More severely depression	3 (18.75)	5 (31.25)	8 (25.00)	
**4th week HDRS N (%)**				
Normal	0 (0.00)	0 (0.00)	0 (0.00)	
Mild	6 (37.50)	5 (31.25)	11 (34.38)	
Moderate	8 (50.00)	8 (50.00)	16 (50.00)	0.700
Severe	2 (12.50)	1 (6.25)	3 (9.38)	
More severely depression	0 (0.00)	2 (12.50)	2 (6.25)	
**8th week HDRS N (%)**				
Normal	13 (81.25)	11 (68.75)	24 (75.00)	
Mild	3 (18.75)	4 (25.00)	7 (21.88)	
Moderate	0 (0.00)	0 (0.00)	0 (0.00)	0.685
Severe	0 (0.00)	1 (6.25)	1 (3.13)	
More severely depression	0 (0.00)	0 (0.00)	0 (0.00)	

Based on the results that were shown in Table 2, a statistically significant difference in the spectrum of depression was not observed between two intervention groups by different study times (P-value > 0.05). Based on the generalized linear estimating equations (GEE) regression test, the HDRS index generally decreased significantly over time in the two intervention groups (P_valuetime < 0.001). On the other hand, the intervention has had a statistical effect on changes in HDRS over time (P-valuetime*group < 0.001). The average FSFI increased during the study in the L-arginine group and decreased over time in the placebo group. But it was not statistically significant (P-value > 0.05).

As seen in the results that were shown in (Table 3), unlike the placebo group, only in the L-arginine group, there were significant incremental changes in the lubrication (*P*-value = 0.032) and orgasm index (*P*-value= 0.023). In comparison with the pre-intervention period, there was a noteworthy shift in the lubrication factor at the fourth and eighth weeks, respectively. Furthermore, it was noted that there was a statistically significant rise in the mean orgasm index throughout the eight weeks of the intervention when compared to the start of the trial (time zero). However, the mean difference between intervention and placebo groups was not statistically significant in any period (P_value > 0.05). Based on the results of (Table 4), in three time periods, the average score of HDRS after the intervention decreased significantly in both groups (P-value < 0.05). However, the mean difference in L-arginine group compared to the placebo group after the interventions was not statistically significant in any of the periods between two groups (P-value > 0.05).

**Table 3:** Changes in mean of HDRS and FSFI and its sub-groups during study between two groups

Index	Groups	Baseline	4^th^ week	8^th^ week	*P*-value time effect	*P*-value time × groups
HDRS (Mean ± SD)	Placebo	15.06 ± 7.05	8.50 ± 4.06	5.62 ± 3.24	<0.001^*^	<0.001^*^
	L-arginine	18.06 ± 8.03	9.75 ± 5.29	5.93 ± 4.87	<0.001^*^	
FSFI (Mean ± SD)	Placebo	22.71 ± 8.98	20.28 ± 11.21	19.83 ± 10.82	0.497	0.189
	L-arginine	17.78 ± 10.96	20.26 ± 11.44	19.92 ± 10.98	0.058	
Desire (Mean ± SD)	Placebo	3.67 ± 2.58	3.11 ± 1.44	3.40 ± 1.22	0.343	0.419
	L-arginine	3.00 ± 1.33	3.03 ± 1.46	2.74 ± 1.16	0.623	
Arousal (Mean ± SD)	Placebo	3.15 ± 1.87	2.73 ± 2.07	2.76 ± 1.92	0.536	0.522
	L-arginine	2.63 ± 2.10	2.93 ± 2.01	2.92 ± 2.05	0.231	
Lubrication (Mean ± SD)	Placebo	3.90 ± 1.76	3.58 ± 2.23	3.31 ± 2.12	0.494	0.137
	L-arginine	2.92 ± 2.23	3.46 ± 2.25	3.50 ± 2.21	0.032^*^	
Orgasm (Mean ± SD)	Placebo	3.90 ± 1.91	3.53 ± 2.25	3.28 ± 2.23	0.474	0.078
	L-arginine	2.63 ± 2.14	3.13 ± 2.21	3.28 ± 2.20	0.023^*^	
Satisfaction (Mean ± SD)	Placebo	3.81 ± 1.88	3.56 ± 2.03	3.65 ± 1.96	0.860	0.311
	L-arginine	3.35 ± 2.04	3.85 ± 2.03	3.78 ± 1.84	0.063	
Pain (Mean ± SD)	Placebo	4.27 ± 1.97	3.75 ± 2.45	3.41 ± 2.35	0.426	0.370
	L-arginine	3.23 ± 2.39	3.83 ± 2.41	3.67 ± 2.36	0.167	

Values described as mean ± standard deviation

*Statistically significant, *P*-value < 0.05 based on Generalized Estimation Equation (GEE) method

**Table 4:** Pairwise comparisons of HDRS and FSFI and its sub-groups in different visit’s times based on interventional groups

Placebo				L-arginine		
Index	Time	Mean difference ± Standard deviation difference¹	Paired comparison, *P*_value^2^	Mean difference ± Standard deviation difference¹	Paired comparison, *P*_value^2^	Between groups comparison^3^
HDRS	T4^th^ - T _baseline_	-6.56 ± 5.99	0.0005*	-8.31 ± 7.99	0.002*	0.533
	T8^th^ - T _baseline_	-9.43 ± 7.50	0.0004*	-12.12 ± 7.39	0.0004*	0.192
	T8^th^ - T4^th^	-2.87 ± 2.70	0.0007*	-3.81 ± 3.25	0.001*	0.382
FSFI	T4^th^ - T _baseline_	-2.43 ± 9.35	0.452	2.48 ± 6.45	0.25	0.374
	T8^th^ - T _baseline_	-2.88 ± 10.22	0.338	2.14 ± 5.25	0.14	0.073
	T8^th^ - T4^th^	-0.44 ± 5.97	0.794	-0.33 ± 8.55	0.697	0.747
Desire	T4^th^ - T _baseline_	-0.56 ± 2.42	0.452	0.03 ± 0.94	0.25	0.843
	T8^th^ - T _baseline_	-0.27 ± 2.53	0.771	-0.25 ± 1.20	0.526	0.564
	T8^th^ - T4^th^	0.28 ± 1.00	0.372	-0.29 ± 1.31	0.812	0.557
Arousal	T4^th^ - T _baseline_	-0.41 ± 1.57	0.303	0.30 ± 1.37	0.397	0.179
	T8^th^ - T _baseline_	-0.38 ± 1.61	0.359	0.29 ± 0.73	0.13	0.141
	T8^th^ - T4^th^	0.03 ± 1.06	0.889	-0.006 ± 1.36	0.985	0.92
Lubrication	T4^th^ - T _baseline_	-0.31 ± 2.33	0.875	0.54 ± 1.25	0.030*	0.438
	T8^th^ - T _baseline_	-0.58 ± 2.32	0.329	0.58 ± 1.36	0.016*	0.05
	T8^th^ - T4^th^	-0.26 ± 1.30	0.514	0.03 ± 1.91	0.65	0.401
Orgasm	T4^th^ - T _baseline_	-0.36 ± 2.37	0.384	0.50 ± 1.44	0.656	0.226
	T8^th^ - T _baseline_	-0.61 ± 2.48	0.334	0.65 ± 1.08	0.030*	0.074
	T8^th^ - T4^th^	-0.25 ± 1.11	0.487	0.15 ± 1.71	0.504	0.352
Satisfaction	T4^th^ - T _baseline_	-0.25 ± 1.98	0.622	0.50 ± 1.26	0.134	0.212
	T8^th^ - T _baseline_	-0.16 ± 2.25	0.777	0.43 ± 0.93	0.079	0.336
	T8^th^ - T4^th^	0.08 ± 1.40	0.29	-0.06 ± 1.43	0.978	0.581
Pain	T4^th^ - T _baseline_	-0.52 ± 2.59	0.356	0.60 ± 1.53	0.076	0.083
	T8^th^ - T _baseline_	-0.86 ± 2.75	0.2	0.43 ± 1.78	0.22	0.095
	T8^th^ - T4^th^	-0.33 ± 1.94	0.663	-0.16 ± 2.33	0.664	0.984

Values described as mean ± standard deviation

^1^Difference = Value after – Value before

^2^Comparison of before intervention value with after intervention value (paired comparison)

^3^Comparison of difference values between two groups of intervention

^*^Statistically significant, *P*_value < 0.05

The effect of intervention on changes in HDRS, FSFI, and its subgroups are shown in Table 5. At the univariate level, the intervention groups had no significant association with the changes in the mentioned indicators (*P*_value > 0.05).

**Table 5:** Results of univariate and multivariable linear generalized estimating equation about effect of intervention on mean changes of HDRS and FSFI and its sub-groups during study

Outcomes	Groups	Model 1 Crude ß^1^, 95% CI	P_value	Model 2 Adjusted ß ^1^, 95% CI	P_value
	Placebo	Reference		Reference	
HDRS	L-arginine	1.52 (-1.44, 4.48)	0.314	0.49 (-1.75, 2.75)	0.666
FSFI	L-arginine	-1.61 (-8.17, 4.93)	0.628	0.37 (-4.99, 5.74)^*^	0.891
Desire	L-arginine	-0.46 (-1.32, 0.38)	0.282	-0.21 (-0.91, 0.48)^*^	0.548
Arousal	L-arginine	-0.05 (-1.30, 1.19)	0.932	0.48 (-0.57, 1.54)^*^	0.371
Lubrication	L-arginine	-0.30 (-1.56, 0.95)	0.638	0.08 (-0.86, 1.03)^*^	0.860
Orgasm	L-arginine	-0.55 (-1.82, 0.72)	0.396	-0.08 (-1.18, 1.02)^*^	0.887
Satisfaction	L-arginine	-0.01 (-1.17, 1.15)	0.983	0.06 (-1.02,1.14)^*^	0.914
Pain	L-arginine	-0.20 (-1.54, 1.08)	0.732	0.04 (-0.96, 1.04)^*^	0.935

^1^Coefficient (ß), 95% Confidence Interval

Model 1: interventional groups

Model 2: age, BMI, interventional groups, underlying diseases, level of education, job status, place of living

*In multivariable model for these outcomes, the HDRS during study time was adjusted

Therefore, after considering the effect of other possible confounding variables, based on the results of multivariable linear GEE regression, no significant statistical association between the type of drug used and the average changes of considered outcomes during the study was observed (*P*_value > 0.05). Although, in general, the average score of FSFI during the study in the L-arginine-consuming group increased by about half a unit compared to the placebo group, these changes were not statistically significant (β = 0.37, 95% CI = -4.99, 5.74, *P*_value = 0.891).

Based on the results of (Table 6), after comparing with the placebo group, there was no significant difference among the complications reported between these two groups (P_value = 0.456).

**Table 6:** Side effects related intervention between L-arginine and placebo groups

Type of side effect *N* (%)	Placebo			
	(***n*= 16)**	(***n*= 16)**	(***n*= 32)**	
Swallowing difficulties (Dysphagia)	1 (6.25)	1 (6.25)	2 (6.25)	
GI upset	0 (0.00)	2 (12.50)	2 (6.25)	
Mastalgia	1 (6.25)	0 (0.00)	1 (3.13)	
Muscular pain	0 (0.00)	1 (6.25)	1 (3.13)	0.456
Hand and foot edema	0 (0.00)	1 (6.25)	1 (3.13)	
No reaction	14 (87.50)	11 (68.75)	25 (78.13)	

## Discussion

This triple-blind, randomized, clinical trial showed that in the patients suffering from depression receiving standard anti-depressant treatment, 2 g per day of L-arginine in the intervention group improved depression and some domains of sexual performance. Based on the FSFI questionnaire, significant changes were observed in two domains of lubrication and orgasm in L-arginine recipients. When compared to the placebo group, this improvement was not statistically significant. The results of this research showed that taking regular antidepressants might significantly lessen the severity of depression. Still, adding L-arginine did not cause a significant difference between L-arginine and the placebo groups. Therefore, an increase was observed in the Rosen Questionnaire or FSFI score, but this increase was not statistically significant. It did not contribute to a positive interpretation of the effectiveness of L-arginine with the dose used in improving depression and increasing sexual function. This research is the first to assess the impact of L-arginine as a supplemental therapy on the sexual functioning of women with Major Depressive Disorder, according to our evaluation. Previously, using L-arginine as a complementary therapy to improve male sexual function was confirmed in various studies [33, 38]. A systematic review and meta-analysis resulted from a randomized clinical trial that included 540 males with erectile dysfunction, using L-arginine with dosing 1500 to 5000 mg effectively improved erectile dysfunction [32]. In a systematic review in 2021, it was found that the compound containing L-arginine called ArginMax can be effective in the treatment of Hypoactive Sexual Desire Disorder and Related Conditions in Women. Five randomized controlled trials and two nonrandomized studies were identified to meet the inclusion criteria based on this systematic review. Six of the seven studies that were included in this had favorable results, indicating either a rise in the overall mean score of the Female Sexual Function Index or a substantial improvement in several areas within the index. Therefore, one study assessed vaginal pulse amplitude and found a statistically significant increase in a combination treatment group compared to placebo. No significant side effects were reported from these studies [39].

One of the most important causes of sexual dysfunction in women is depression, which has been proven in various studies [40, 41]. Consequently, it is anticipated that sexual function would increase in these individuals with well-managed and treated depression. However, some people have sexual dysfunction as a result of using certain drugs to treat depression.

One of the most important drugs for treating Major Depressive Disorder is an SSRI. One of the side effects of SSRIs is sexual dysfunction. Based on some studies, SSRIs-induced sexual dysfunction was observed in 25 to 73% of the patients who use these medications for depression treatment [22]. The interesting point is that this property of SSRIs is used to treat premature ejaculation disorder. Based on a meta-analysis in 2021, SSRI treatment probably improves self-perceived premature ejaculation symptoms [42]. The patients were first treated with either sertraline or escitalopram, and intervention in treating patients using L-arginine or placebo began at the end of the fourth week. Based on the scores obtained from the FSFI questionnaire, in the placebo group, the scores of the patients throughout the study had a decreasing trend, which indicated the sexual complications caused by SSRI medication. Unlike the placebo group, in the patients receiving L- arginine, the FSFI score increased in the eighth week compared to the beginning of the study (P-value = 0.058). Although this increase in scoring compared to the placebo group was not statistically significant, it showed that adding L-arginine to the standard antidepressant treatment of patients can positively affect sexual performance. The impact of SSRIs in creating sexual dysfunction may be linked to the absence of statistically meaningful response in patients when L-arginine was used to improve sexual performance. It seems that L-arginine cannot improve SSRI-induced sexual dysfunction, as statistically significant. In previous studies where L-arginine has shown statistically significant effects on sexual function, the recipients of this agent have no mental disorder and use SSRI medications to treat mental illness. In addition to SSRI-induced sexual dysfunction, the lack of statistical significance of the addition of L-arginine on sexual performance despite the improvement of the FSFI score in the L-arginine group may be that in terms of its lower doses compared to most of the other studies [39]. Among the subdomains of FSFI, the lubrication index showed a significant increase in the L-arginine group in the eighth week compared to the baseline level. It should be noted that this rise was not statistically significant when compared to the placebo group. Additionally, the eighth week of the L-arginine group exhibited a substantial change in the orgasm index compared to the baseline level, however this difference was not statistically significant when compared to the placebo group. Although these changes were not statistically significant, they can create a suitable perspective for further research to obtain better results by removing the limitations of this study, which were mentioned earlier. These findings are consistent with previous studies that showed that using L-arginine in the women can positively affect orgasm and lubrication [43]. However, the increase in sexual desire in women, observed in most of the studies mentioned above following the consumption of L-arginine, was not seen in this study, even compare to different periods in the L-arginine group.

Based on a systematic review in 2023, L-arginine and L-lysine are effective therapeutic interventions to reduce anxiety and mental stress [44]. Although some animal studies have suggested that stress, and the resulting brain damage that can lead to depression will be reduced with L-arginine, there are few human studies in this regard [45]. The impact of incorporating L-arginine into the conventional antidepressant therapy, which includes one of the two medications, escitalopram or sertraline, was assessed in this study due to the paucity of research on the efficacy of this amino acid in treating depression. Although using of standard depression treatment could significantly reduce the severity of depression in patients of both groups, adding L-arginine to the treatment regimen of patients did not have a statistically significant effect.

Because of genetic differences, laboratory evaluation of L-arginine levels, and its metabolites in the patients may be a guide to better patient assessment. If the levels of different metabolites of L- arginine were evaluated in the patients from the beginning of the study, they could show a better view of the treatment process. Major depressive disorder may arise as a result of variations in the urea and nitric oxide cycles’ concentrations of arginine and its associated catabolic products, including ornithine, citrulline, and argininosuccinate. It seems that conducting an integrated assessment of arginine, and its related catabolic products could potentially aid in predicting the probability of developing Major Depressive Disorder [44].

As previous studies showed, using L-arginine has no significant patient side effects. The study conducted by D Menafra et al. aimed to investigate the effects of L-arginine on penile erectile function in patients with vasculogenic erectile dysfunction. The study was multicenter, double- blind, randomized, and placebo-controlled, with a total of 98 participants (51 on L-arginine and 47 on placebo), and was published in 2022. The participants received a relatively high daily oral dose of 6 g of L-arginine for three months. The study compared the outcomes of mild- moderate and severe vasculogenic erectile dysfunction. The results of the study showed no serious complications reported in patients who received L-arginine [46]. In our study, the patients reported no dangerous side effects or injuries. The only side effect reported in the patients, slightly more than in the placebo group, was gastrointestinal upset. However, these side effects were not statistically significant compared to the placebo group.

## Limitations

The Iranian pharmaceutical market lacked high-dose L-arginine products. Consequently, to improve the patient compliance, the minimum effective dose established in prior studies was necessarily used.

Despite achieving the minimum required sample size, and effectively implementing the research design, cultural considerations in Iran presented unexpected recruitment challenges, limiting our ability to further increase participant numbers. Furthermore, the 8-week study timeframe likely discouraged participation in a potentially longer treatment period, making extension impractical.

## Conclusion

With little side effects reported, supplementing with L-arginine may enhance mood and sexual function, especially lubrication and orgasm, in women experiencing depression. Future studies investigate the effect of higher doses, longer durations, and larger populations of depressed patients are warranted to solidify these findings.

## Data Availability

The datasets generated during and/or analyzed during the current study are available from the corresponding author on reasonable request.

## Abbreviations

MDD: Major depressive disorder
HDRS: Hamilton depression rating scale
FSFI: Female sexual function index
SSRIs: Selective serotonin reuptake inhibitors
GEE: Linear generalized estimating equations
